# Removal of EMG Artifacts from Multichannel EEG Signals Using Combined Singular Spectrum Analysis and Canonical Correlation Analysis

**DOI:** 10.1155/2019/4159676

**Published:** 2019-12-30

**Authors:** Qingze Liu, Aiping Liu, Xu Zhang, Xiang Chen, Ruobing Qian, Xun Chen

**Affiliations:** ^1^Department of Electronic Science and Technology, University of Science and Technology of China, Hefei 230027, China; ^2^Department of Neurosurgery, The First Affiliated Hospital of University of Science and Technology of China (Anhui Provincial Hospital), Hefei 230036, China; ^3^Department of Electronic Engineering and Information Science, University of Science and Technology of China, Hefei 230026, China

## Abstract

Electroencephalography (EEG) signals collected from human scalps are often polluted by diverse artifacts, for instance electromyogram (EMG), electrooculogram (EOG), and electrocardiogram (ECG) artifacts. Muscle artifacts are particularly difficult to eliminate among all kinds of artifacts due to their complexity. At present, several researchers have proved the superiority of combining single-channel decomposition algorithms with blind source separation (BSS) to make multichannel EEG recordings free from EMG contamination. In our study, we come up with a novel and valid method to accomplish muscle artifact removal from EEG by using the combination of singular spectrum analysis (SSA) and canonical correlation analysis (CCA), which is named as SSA-CCA. Unlike the traditional single-channel decomposition methods, for example, ensemble empirical mode decomposition (EEMD), SSA algorithm is a technique based on principles of multivariate statistics. Our proposed approach can take advantage of SSA as well as cross-channel information. The performance of SSA-CCA is evaluated on semisimulated and real data. The results demonstrate that this method outperforms the state-of-the-art technique, EEMD-CCA, and the classic technique, CCA, under multichannel circumstances.

## 1. Introduction

As a representatively noninvasive technique of reflecting electrical activities generated by the cerebral cortex, electroencephalography (EEG) is widely used for numerous practical applications in the biomedical engineering field. It owns the benefits of low cost, easy usability, and high temporal resolution. For example, EEG recordings are important for the description of the irritant and ictal onset zones in the presurgical evaluation of refractory partial epilepsy [1]; motor imagery EEG signals provide an important basis for designing a way to communicate between the brain and computer [2]; by making use of sparse EEG compressive sensing, person identification is possible [3]; and EEG can be utilized with other physiological data of different types to make a study of brain functions [4]. Nevertheless, with relatively low amplitudes, EEG is often polluted by many kinds of nonbrain artifacts mainly from the electromyogram (EMG), electrooculogram (EOG), and electrocardiogram (ECG) interferences. Thus, it is difficult to continue subsequent signal analysis. If the pollution is very heavy, the EEG waves may be completely masked so that we cannot interpret the brain activity contained in EEG signals [5]. Therefore, it has been attracting increasing attention that how to effectively eliminate these artifacts in the last few decades [6, 7].

Compared with EOG and ECG artifacts, there are more troubles in the domain of removing EMG artifacts [8, 9]. As we all know, many kinds of movements involving but not limited to eye movement, mastication, and facial expression are generated by a number of muscles around the head. EEG can be easily influenced anywhere on the human scalp by the activity of each muscle via volume conduction. EMG artifacts have the characteristics of high amplitude, nonstereotyped scalp topographies, and extensive frequency domain distributions, which increase the difficulty in denoising.

In previous studies, researchers have successfully explored blind source separation (BSS) approaches to handle multichannel EEG data for accomplishing artifact elimination. Both independent component analysis (ICA) and canonical correlation analysis (CCA) belong to the most classic methods. ICA exploits higher-order statistics (HOS) of data to decompose the multichannel signals into independent components (ICs). The ICs representing the underlying sources are reserved, but artifact-related ICs are identified and discarded; hence, we can reconstruct relatively artifact-free EEG subsequently. As a well-known and effective BSS method, ICA is widely adopted for artifact removal from EEG since its first application in the field of brain electrical noise reduction [10, 11]. A study comparing fifteen diverse algorithms of ICA for denoising muscle artifacts has been published, which provides us with a helpful reference [12]. However, when it comes to the EMG removal problem of EEG, ICA might not perform well [9]. By making use of HOS information, ICA is better at eliminating the artifacts whose scalp topographies are stereotyped such as EOG. Muscle artifacts usually own a lot of different scalp topographies involving the activities of a group of muscles. Moreover, ICA does not exploit the temporal structure of muscle activities. Later on, canonical correlation analysis (CCA) has been advised to achieve EMG artifact removal [13]. By utilizing second-order statistics (SOS) information, CCA is able to obtain sources which are autocorrelated to the great extent and mutually uncorrelated. Since EMG artifacts have a broad frequency spectrum, their autocorrelation is low while the autocorrelation of EEG rhythms is high relatively. CCA can utilize this obvious characteristic to eliminate EMG artifacts. Simulation studies [13–15] and clinical studies [16, 17] have proved the superiority of CCA beyond ICA frequently for suppressing muscle artifacts in EEG.

However, with low signal-to-noise ratio (SNR) and complex contamination, ICA and CCA cannot perform well enough when denoising noisy EEG [6, 15]. As application scenarios of EEG devices tend to change from the traditional experimental condition to the realistic complex dynamic environment, muscle artifacts are inevitably generated due to the head movement and they are often pretty heavy. In recent years, studies show that combining more than one algorithm might obtain optimal results for removing artifacts from the EEG signals [7, 18, 19]. Usually, combining single-channel decomposition methods with BSS is highly recommended.

It is common that the combination mentioned above is applied to process single-channel EEG [20]. Since BSS implicitly has the limitation that potential sources must be not more than utilizable channels in number, the single-channel EEG can be decomposed into multidimensional data sets by single-channel decomposition methods to satisfy the requirements of BSS. For example, the combination of the wavelet transform (WT) with ICA [21], ensemble empirical mode decomposition (EEMD) with multiset CCA (MCCA) [22], and so on. In order to eliminate muscle artifacts from multichannel EEG recordings, if we process the multichannel EEG by means of channel by channel using the combination mentioned above, the relationship between channels may be ignored. To overcome this shortcoming, an EEMD-ICA approach has been suggested to improve the artifact elimination effect for multichannel EEG signals [23]. The EEMD-ICA approach employs ensemble empirical mode decomposition (EEMD) [24] to firstly obtain a number of intrinsic mode functions (IMFs) from each channel of EEG data. Then, the acquired IMFs relating to EMG artifacts are selected according to predefined rules. When applied to the chosen IMFs, ICA has the ability of concentrating the contents involving artifacts into several specific ICs. By discarding the ICs related to artifacts, we can obtain the relatively artifact-free data ultimately in the reconstruction step. It has been proved that this EEMD-ICA approach outperforms ICA and wavelet-ICA [25]. The superior performance over ICA demonstrates that exploring the information of every signal channel by single-channel decomposition methods first is of great significance to the contaminated multichannel EEG. The superior performance over wavelet-ICA is not hard to explain. While wavelet transform (WT) decomposes a signal adopting the method of determining in advance, and it is not easy in practice to select the best mother wavelets, EEMD is an entirely data-driven method, where no prior knowledge is required during decomposition. As we have discussed, CCA is better suited to eliminate muscle artifacts with complex and multiform scalp topographies. Very recently, replacing ICA with CCA, the EEMD-CCA method outperforms different techniques, including ICA, CCA, and EEMD-ICA, for eliminating muscle artifacts from EEG with multiple channels [26]. As far as we know, it gets the best results on multichannel EEG denoising. But EEMD decomposes the signal merely in terms of the amplitude and frequency information [15], and it cannot separate EEG contents from EMG artifacts overlapping in the relatively higher-frequency band. The IMFs relating to EMG artifacts usually represent high-frequency bands. Since the amplitude of EEG contents in the IMFs mainly containing muscle artifacts is much lower than the amplitude of EMG artifacts, it is extremely difficult to extract brain activity drowned in artifacts. Therefore in this article, we utilize singular spectrum analysis (SSA) to accomplish single-channel decomposition and propose a new architecture to process multichannel EEG data.

Singular spectrum analysis (SSA) is a kind of spectrum estimation technique with no need for parameters to do the decomposition for the raw signal according to the covariance property of data [27] and the characteristic of original signal [28]. In addition to its great success in terms of handling climatic, meteorological, and geophysical data [29], the SSA-based algorithm has been used to analyse EEG signals. Maddirala and Shaik made use of the method based on SSA to eliminate EOG [30] and motion artifacts [31] from EEG in the case of only one channel; Teixeira et al. presented an approach to extract high-amplitude artifacts [32]; Hu et al. suggested utilizing the method derived from SSA to extract desired brain rhythms [33]. On the basis of these studies, we know that SSA can succeed in separating EEG composed of different sources, which are mixed with each other in the time-frequency domain. Cheng et al. have demonstrated that SSA is more powerful than EEMD in decomposing single-channel EEG [34].

In our proposed method, with the goal of eliminating EMG artifacts from multichannel EEG data, SSA algorithm is utilized twice. It decomposes each channel of multichannel EEG signals to acquire a collection of interpretable components. The two data sets of relatively clean EEG reconstructed from the process of two-time SSA decomposition are handled with CCA to get further noise reduction. Here, we set a suitable threshold value for the autocorrelation to select the components containing EEG information automatically. Our proposed SSA-CCA approach is applied to semisimulated data and real-life data, respectively; meanwhile, we make a comparison with the most effective technique, EEMD-CCA, and the classic technique, CCA.

The main contribution of our study is that we successfully solved the problem for removing EMG artifacts from EEG data in the multichannel situation. Our proposed technique is novel and performs very well. This scheme not only takes advantage of SSA to conduct time series analysis better than EEMD, but also utilizes a new framework to seek crosschannel interdependence with the help of BSS. It is also novel that we distinguished components of different content types by calculating the autocorrelation coefficients of SSA components.

The organization of the remaining paper is as follows: the proposed method and the methods used to do the comparison are described in Section 2. In Section 3, the synthetic and real-life data are briefly introduced.  Section 4 presents the denoising results.  Section 5 offers an intensive discussion and summarizes the work in this paper in the end.

## 2. Methods

We will introduce the suggested SSA-CCA method and the methods used to compare, i.e., CCA and EEMD-CCA in the following text of this section. The notations will be employed throughout the article as follows: lowercase italic letters (*x*, *y*,…) are on behalf of scalars, lowercase boldface letters (**x**, **y**,…) take the place of vectors, boldface capitals (**X**, **Y**,…) represent matrices, and furthermore italic capitals (*C*, *T*,…) are on behalf of the number of rows and columns. Vector or matrix transposition can be represented by the uppercase superscript *T* (e.g., **x**^*T*^ as well as **Y**^*T*^). In our study, the multichannel EEG signal is written as a matrix **X** whose size is *C* × *T*. It means that this signal includes *C* channels and *T* sampling points. The time course is represented as **X**(*t*)=[**x**_1_(*t*), **x**_2_(*t*),…,**x**_*C*_(*t*)]^*T*^ (*t*=1,2,…, *T*).

**Figure 1:**
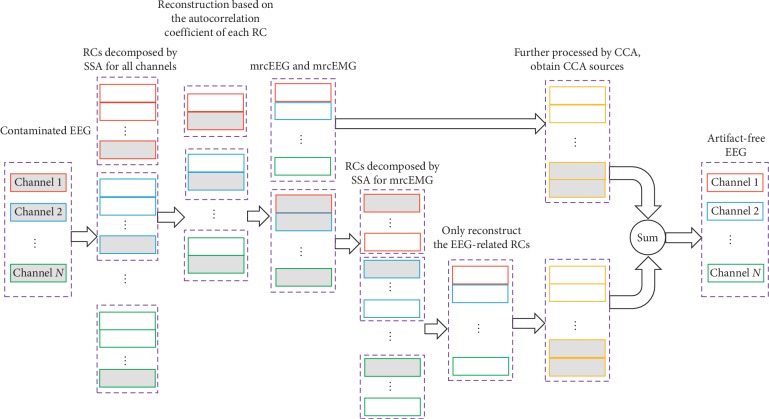
A flow diagram for the SSA-CCA approach. Here, the grey rectangles represent artifact-related components.

The entire flow diagram of the suggested SSA-CCA scheme is as shown in Figure 1. As it can be seen, SSA-CCA contains the following five steps: (1) utilizing SSA to decompose each channel into a collection of reconstructed components (RCs); (2) selecting RCs related to EEG activity to reconstruct a multichannel relatively clean EEG, mrcEEG, and the multichannel relatively clean EMG, mrcEMG, in the meanwhile; (3) decomposing mrcEMG by SSA again to extract possible EEG content just like step (1) and step (2); (4) applying CCA to the two data sets of mrcEEG from step (1) and step (3) for removing artifacts and obtaining cleaned mrcEEG; and (5) adding the two cleaned mrcEEG data up to get the desired artifact-free EEG. The description of details about our proposed approach is provided as follows.

### 2.1. Decomposition of Each Channel Using SSA

SSA is a very effective approach for analyzing time series. Considering a *N* sampled signal of one dimension as **x**(*t*)=(*x*_1_, *x*_2_,…, *x*_*N*_), the SSA algorithm is composed of two stages, which are named as decomposition and reconstruction, respectively. There are also two independent steps at each stage. To be more specific, the decomposition stage involves time-delay embedding and singular value decomposition (SVD). First, when conducting the embedding step, the original one-dimensional signal **x** can be mapped into a trajectory matrix **X** whose size is *L* × *K*:

(1) $$\begin{array}{c} X=\left(x_{1},x_{2},\ldots ,x_{K}\right)=\left[\begin{array}{cccc} x_{1} & x_{2} & \cdots & x_{K}\\ x_{2} & x_{3} & \cdots & x_{K+1}\\ \vdots & \vdots & \ddots & \vdots \\ x_{L} & x_{L+1} & \cdots & x_{N} \end{array}\right], \end{array}$$

where *L* is on behalf of the window length for segmenting the data and **x**_*i*_(1 ≤ *i* ≤ *K*) denotes the lagged vector as one column in the matrix **X** and here, we define *K*=*N* − *L*+1. In the obtained trajectory matrix, all the antidiagonal elements are the same, and the matrix of this type is called Hankel matrix. Then, with the purpose of calculating SVD of matrix **X**, the SVD step can be accomplished by utilizing the eigenvalue decomposition (EVD) of the covariance matrix **C**, where **C**=**XX**^*T*^. Here, all eigenvalues and eigenvectors of the covariance matrix **C** are denoted as *λ*_1_, *λ*_2_,…, *λ*_*L*_ and **v**_1_, **v**_2_,…, **v**_*L*_, respectively. It must be pointed out that the eigenvalues and corresponding eigenvectors are sorted inherently following the order of magnitude decrease, i.e., *λ*_1_ ≥ *λ*_2_ ≥ ,…, ≥*λ*_*L*_ ≥ 0. The elementary matrices are defined as

(2) $$\begin{array}{c} X_{i}=\sqrt{\lambda_{i}}v_{i}u_{i},\;\left(i=1,2,\ldots ,L\right), \end{array}$$

where $u_{i}=X^{T}v_{i}/\sqrt{\lambda_{i}}$. Now, the trajectory matrix **X** can be expressed as

(3) $$\begin{array}{c} X=\overset{L}{\underset{i=1}{\sum }}X_{i}=\overset{L}{\underset{i=1}{\sum }}\sqrt{\lambda_{i}}v_{i}u_{i}. \end{array}$$

Reconstruction consists of grouping and diagonal averaging. The SSA grouping involves dividing the indices *I*_*n*_=1,2,…, *L* into *G* different groups. The trajectory matrix after the grouping step is denoted by

(4) $$\begin{array}{c} X=\overset{G}{\underset{j=1}{\sum }}X_{I_{j}}, \end{array}$$

where **X**_*I*_*j*__ is the trajectory matrix and *I*_*j*_ is an ensemble of indices for the *j*^th^ group, *j*=1,…, *G*. In the condition of *G*=*L*, grouping of this type is known as elementary grouping. We conduct elementary grouping in this paper, i.e., *j*=1,…, *L*. In the final step called diagonal averaging since each submatrix **X**_*I*_*j*__ is hankelized, we can transform the acquired Hankel matrix into a new series through the operation of changing the antidiagonal elements of the matrix. The element values on the opposite diagonal of the Hankel matrix will be replaced with their mean value, which will be used to generate the signal of one dimension later. The reconstructed time series are referred to as reconstructed components (RCs) or SSA components in general. Thus, the original *N* sampled signal **x**(*t*) can be presented by

(5) $$\begin{array}{c} x\left(t\right)=\overset{L}{\underset{j=1}{\sum }}x_{j}^{\text{rc}}\left(t\right), \end{array}$$

where **x**_*j*_^rc^(*t*) denotes the *j*^th^ RC with elementary grouping.

### 2.2. RCs Selection

According to our introduction to the SSA decomposition principle, it can be seen that there is no difference between choosing indices one wants at the grouping step and selecting proper RCs after elementary grouping while extracting a desired signal. However, there is no general criterion for indices or RCs selection [35]. When the energy of our expected signal is high enough and the signal that we need can be well defined in advance, the minimum description length (MDL) criterion works well for automatic grouping of trajectory matrices according to the magnitude of the eigenvalues [32]. Besides, in order to extract the dominating rhythms contained in EEG, researchers group the eigenvectors by exploring the characteristic of the eigenvalue pairs [36].

As we all know, muscle artifacts have a wide spectrum in the frequency domain and they behave very much like white noise, whose autocorrelation is much lower in comparison. The group rules above have a bad performance when applied to EMG artifact removal. Here, we recommend calculating the autocorrelation coefficient of each RC. The autocorrelation coefficient is a widely adopted indicator for the muscle artifact removal issue to select artifact-related components generated by EEMD [26]. Picking out RCs with relatively higher autocorrelation values can ensure that the useful information of EEG signals is reserved. We set an appropriate threshold value for the autocorrelation to identify and pick out RCs related to EEG rhythms before reconstruction automatically. Suppose one RC is represented as **c**(*t*), let **c**_1_(*t*) equal to **c**(*t*) and **c**_2_(*t*) be the time-delayed version, i.e., **c**_2_(*t*)=**c**(*t* − 1). The calculation formula of autocorrelation *R* is as follows:

(6) $$\begin{array}{c} R=\frac{E\left[\left(c_{1}\left(t\right)-E\left(c_{1}\left(t\right)\right)\right)\left(c_{2}\left(t\right)-E\left(c_{2}\left(t\right)\right)\right)\right]}{\sqrt{E\left[{\left(c_{1}\left(t\right)-E\left(c_{1}\left(t\right)\right)\right)}^{2}\right]E\left[{\left(c_{2}\left(t\right)-E\left(c_{2}\left(t\right)\right)\right)}^{2}\right]}}, \end{array}$$

where *E* is the operator that computes the expectation.

The RCs with autocorrelation values less than the threshold are picked out to generate relatively clean EEG. When one has finished this process channel by channel, the multichannel relatively clean EEG, denoted as mrcEEG, is obtained. And the multichannel relatively clean EMG, denoted as mrcEMG, can be obtained by subtracting mrcEEG from the original mixed EEG. Here, a relatively lower threshold is suggested in order to avoid too much loss of brain activity information.

### 2.3. Further Treatment of Muscle Artifact with CCA

The mrcEEG is processed by CCA to achieve further artifact elimination. Let **Z**_1_(*t*) be equal to the mrcEEG matrix **Z**(*t*), which includes *C* channels and *T* sampling points, meanwhile **Z**_2_(*t*) be the time-delayed version, i.e., **Z**_2_(*t*)=**Z**(*t* − 1). CCA maximizes the correlation coefficient (CC) between the related sources generated from **Z**_1_(*t*) and **Z**_2_(*t*). This results in an objective function as follows, which aims at strengthening the correlation as best as it can between the mixtures of the variates from **Z**_1_ and **Z**_2_:

(7) $$\begin{array}{c} \underset{{\text{w}}_{1},{\text{w}}_{2}}{\max}\frac{w_{1}^{T}\sum_{12}w_{2}}{\sqrt{w_{1}^{T}\sum_{11}w_{1}}\sqrt{w_{2}^{T}\sum_{22}w_{2}}}, \end{array}$$

where ∑_11_ the autocovariance matrices of **Z**_1_, ∑_22_ is the autocovariance matrices of **Z**_2_, and ∑_12_ is the crosscovariance matrix of **Z**_1_ and **Z**_2_, in addition **w**_1_ and **w**_2_ are the weight vectors. In our definition, ${\tilde{S}}_{1}$ denotes the whole canonical variates generated from **Z**_1_ and ${\tilde{S}}_{2}$ denotes those generated from **Z**_2_. Traditionally, CCA is considered as a BSS technique by making the estimated sources highly correlated between ${\tilde{S}}_{1}$ and ${\tilde{S}}_{2}$ and mutually uncorrelated within each respective matrix. By this means, the rows in ${\tilde{S}}_{1}$ are arranged in the decreasing order of autocorrelation. In comparison to EEG content, the autocorrelation of EMG artifacts is relatively lower. Therefore, CCA has the ability to concentrate these artifacts into the last several sources. Finally, we can set sources relating to artifacts to zero in the reconstruction step to achieve further artifact elimination.

### 2.4. Artifact Removal and Signal Reconstruction

In this part, by letting the artifact-related sources to be zero and operating the inverse process of CCA, the denoised mrcEEG data can be acquired. Then, the mrcEMG in Section 2.2 should be processed the same way as the original mixed EEG using the combination of SSA and CCA. We find that twice is enough for applying this combination. Finally, add the two cleaned mrcEEG data up. The desired artifact-free multichannnel EEG is done.

### 2.5. Introduction to State-of-the-Art Methods

#### 2.5.1. CCA for Muscle Artifact Elimination

The CCA method has been described in Section 2.3. One can also consult the original work [13].

#### 2.5.2. EEMD-CCA Method for Muscle Artifact Elimination

First, there is an introduction to EEMD. Empirical mode decomposition (EMD), as one well-known decomposition method firstly suggested by Huang et al., is suitable to process many kinds of time variable and complex signals. EMD can decompose a one-dimensional signal into a number of intrinsic mode functions (IMFs), refer to [37] for decomposition details. A single-channel signal **x**(*t*) can be decomposed in the form of

(8) $$\begin{array}{c} x\left(t\right)=\overset{N}{\underset{j=1}{\sum }}c_{j}+r_{n}, \end{array}$$

where **c**_*j*_ denotes the *j*^th^ IMF, *j*=1,2,…, *N* and **r**_*n*_ denotes the residual component after extracting all *N* IMFs. But the original EMD algorithm has its own inherent disadvantage. It is easily influenced by noise, and the phenomenon of mode-mixing is possible to occur among diverse IMFs. Therefore, Wu and Huang [24] came up with a noise-assisted method for data analysis, known as ensemble EMD (EEMD), to solve this problem. EEMD independently adds white noise to the raw signal with a number of individual trials when applying the original EMD. At last, EEMD takes the mean of a collection of IMFs as the definition of its IMFs. In this paper, we have tried different ensemble numbers when utilizing EEMD (i.e., 10, 50, and 100) and there is no significant difference when the number of ensembles is not less than 50. Considering the computational cost and to get the best possible result, 50 ensembles were used. The noise standard deviation was determined as 0.2 times the standard deviation of the raw data according to experience as recommended [24]. The implementation of EEMD-CCA includes six steps. The details of this algorithm are provided in the work [26].

## 3. Data Generation and Description

In order to conduct performance evaluation of different techniques appearing in this article, we made use of semisimulated data and real-life data. The semisimulated EEG signals are derived by mixing real pure EEG with pure EMG, both collected from different subjects. The real-life data set comes from a patient suffering from epileptic seizures. The details of these data are shown as follows.

### 3.1. Semisimulated Data

The semisimulated data set was generated from real EEG and EMG data, which were derived from different people. The 19-channel pure EEG data were recorded when 20 subjects in good health participated in the experiment, whose sampling rate was 500 Hz and processed by a high-passed filter with 1 Hz cutoff frequency to eliminate the baseline noise. The original EMG signals acting as muscle artifact sources were collected with 23 healthy volunteers involved, whose sampling rate was also 500 Hz to match with EEG data. The length of data is 10 seconds for both types of data. The instrument and details of data acquisition can be found in [26].

**Figure 2:**
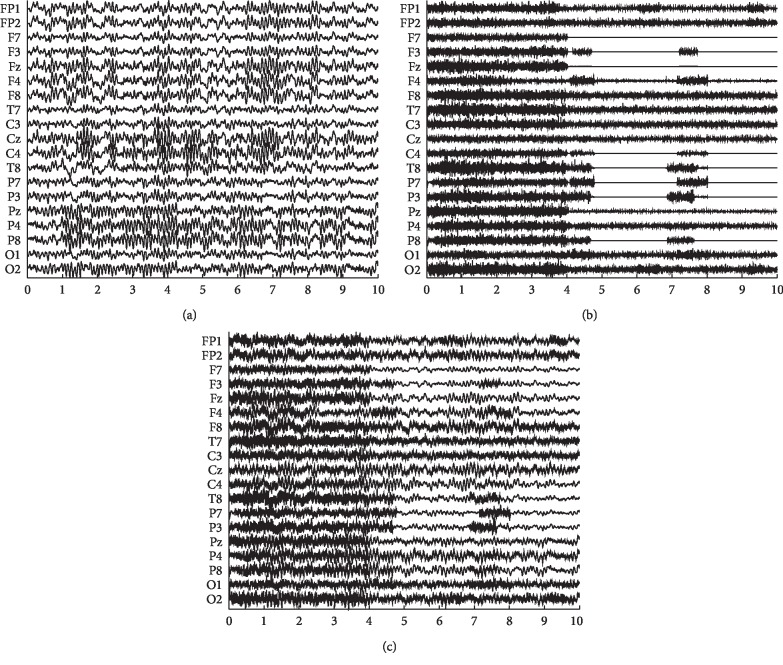
(a) Pure EEG data **X**_EEG_, (b) pure simulated EMG data **X**_EMG_, and (c) mixed EEG data **X** contaminated with SNR = 1.5. The horizontal axis is on behalf of time variation with the second as the unit.

To make sure that the sources were independent with each other and randomly chosen, each EMG source was selected among diverse EMG recordings across different subjects. Thus, an independent EMG source matrix **S**_EMG_ could be formed, and it includes 19 channels with 10 seconds of data in each channel. By multiplying a 19 × 19 mixing matrix **A** with the EMG source matrix **S**_EMG_, a simulated EMG matrix **X**_EMG_ containing 19 channels was generated. To ensure sufficient spatial structure, there were 5 to 8 nonzero elements in each column of the matrix **A**. Thus, each EMG source from the source matrix **S** synchronously exists in 5 to 8 channels of the simulated EMG matrix **X**_EMG_. The number, values, and positions of nonzero entries in each column of the matrix **A** were decided at random on the basis of uniform distribution [6]. At last, the data generated above were utilized to form the mixed EEG signal **X** as follows:

(9) $$\begin{array}{c} X=X_{\text{EEG}}+\lambda \cdot X_{\text{EMG}}, \end{array}$$

where *λ* determines the contribution of EMG artifacts. The contamination levels can be controlled by adjusting the SNR values. SNR is calculated as

(10) $$\begin{array}{c} \text{SNR}=\frac{\text{RMS}\left(X_{\text{EEG}}\right)}{\text{RMS}\left(\lambda \cdot X_{\text{EMG}}\right)}, \end{array}$$

where we define the root mean square (RMS) as

(11) $$\begin{array}{c} \text{RMS}\left(X\right)=\sqrt{\frac{1}{C\cdot T}\overset{C}{\underset{c=1}{\sum }}\overset{T}{\underset{t=1}{\sum }}X^{2}\left(c,t\right)}, \end{array}$$

where *C* represents how many channels there are and *T* represents how many time sampling points there exist. The values of SNR were within a range from 0.5 to 4.5, which are changed taking 0.5 as the step length. As an example, data of pure EEG, pure simulated EMG, and mixed EEG are presented in Figure 2.

In our study, two evaluation indexes were adopted for the semisimulated experiment. The first evaluation indicator was called the relative root mean squared error (RRMSE), which was expressed as

(12) $$\begin{array}{c} \text{RRMSE}=\frac{\text{RMS}\left(X_{\text{EEG}}-{\tilde{X}}_{\text{EEG}}\right)}{\text{RMS}\left(X_{\text{EEG}}\right)}, \end{array}$$

where ${\tilde{X}}_{\text{EEG}}$ is the EEG data after being processed to eliminate artifacts. The second evaluation measure was the correlation coefficient (CC) between the pure EEG served as ground truth in each channel and its denoised version. We calculated the average CC (ACC) values over all channels to estimate the ability of the methods for preserving true brain activity. Note that these two indicators were applied to mean-removed signals.

### 3.2. Real Data

**Figure 3:**
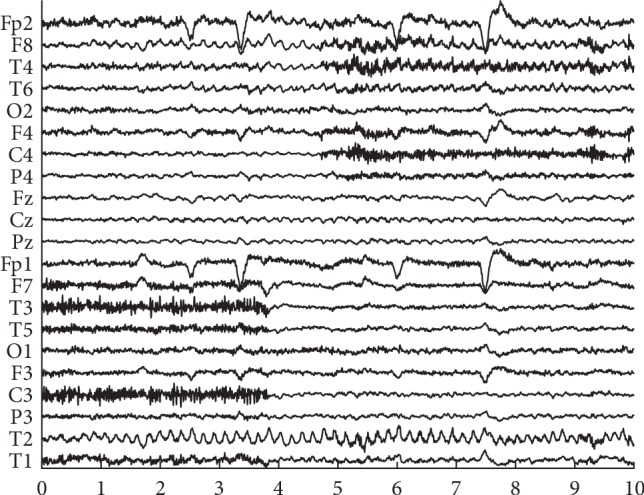
Real EEG data polluted by muscle artifacts. The horizontal axis is on behalf of time variation with the second as the unit.

An available real-life ictal EEG recording was utilized to evaluate the performance between our proposed method and the other two methods on data of the real person. As shown in Figure 3, these data are scalp EEG signal, which contains 21 channels, lasts for 10 s, and has a sampling rate of 250 Hz. It was processed by a band-pass filter from 0.3 to 35 Hz. Muscle artifacts can be found in channels F7, T3, T5, C3, and T1 between 0 and 4 s and in channels F8, T4, F4, C4, and P4 between 5 and 10 s.

Since real data lacks the ground truth to serve as a reference, both RRMSE and ACC cannot be utilized to perform evaluation. Power spectral density (PSD) is an effective and well-known way to describe the energy distribution of time series in the frequency domain. Different from muscle artifacts, the EEG components are mainly at a lower frequency. Hence, the values of PSD belong to a well-denoised EEG signal which have a tendency to decrease at high frequencies (e.g., above 30 Hz) and at the same time they follow more closely the PSD values of the raw EEG signal at low frequencies (e.g., below 25 Hz).

## 4. Results

### 4.1. Semisimulated Data

To make a quantitative comparison, the semisimulated data were handled by all the methods in this paper to automatically eliminate EMG artifacts. There were a total of 20 collected EEG recordings generated from 20 different subjects. In order to sufficiently utilize each EEG recording, 10 independent results were obtained by adding 10 respective simulated EMG matrices, which were randomly produced by the recorded EMG signals. Thus, there were 200 mutually independent results in all per SNR value, and the mean as well as standard deviation at the corresponding SNR value were calculated. According to the method description in Section 2, CCA can isolate the final obtained components related to muscle artifacts into the last several components. Since we own the ground truth here, all the methods will definitely receive their best performance by discarding the optimal number of components at each SNR value. The meaning of the optimal number is that removing fewer or more last components cannot get better results than removing the last components of this number.

**Figure 4:**
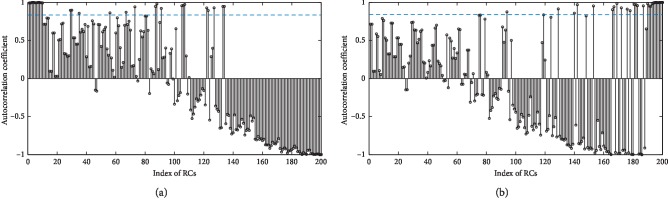
(a) The autocorrelation coefficients of RCs for the original mixed EEG signal at SNR 1, and (b) the autocorrelation coefficients of RCs for the mrcEMG. The blue line is threshold 0.82.

The window length *L* for SSA is empirically recommended as 200. At the SNR value of 1, after the raw mixed EEG signal is decomposed by SSA, the autocorrelation coefficients of reconstructed components (RCs) are shown in Figure 4(a). We recommend setting the threshold to be 0.82. The RCs whose autocorrelation coefficients are above the threshold are selected to reconstruct multichannel relatively clean EEG, mrcEEG. The left RCs generate multichannel relatively clean EMG, mrcEMG. Then, mrcEMG is also decomposed by SSA and the autocorrelation coefficients of RCs are described in Figure 4(b). Now, the RCs related to EEG are isolated relatively behind. With eigenvalues and the corresponding RCs arranged in the decreasing order of magnitude, according to reference [38], the SSA algorithm can be regarded as a bank of finite impulse response (FIR) filters. The filters are data adaptive, and the filter corresponding to the higher-energy component is located in the relatively front position of the RC sequence. In Figure 4(b), the sum of eigenvalues corresponding to EEG-related RCs accounts for 3.13% of the sum of all eigenvalues, which means that after picking out EEG-related RCs, the reconstructed EMG signal contains almost no EEG information. Thus, there is no need to further decompose the EMG signal reconstructed from mrcEMG, considering the time cost of the SSA algorithm.

**Figure 5:**
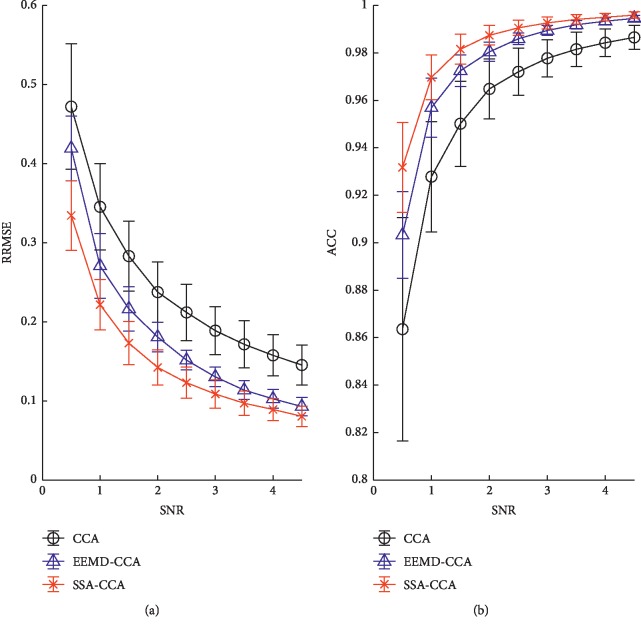
For the semisimulated experiment, the quantitative comparison of methods per different SNR value of (a) RRMSE and (b) ACC.

The final obtained results are displayed in Figure 5. The threshold of EEMD-CCA at step (2) in Section 2.5.2 is 0.95 as recommended [26]. It can be seen that SSA-CCA performs best per SNR value in terms of RRMSE and ACC. EEMD-CCA has a better performance than CCA, reproducing the results in [26].

### 4.2. Real-Life Data

**Figure 6:**
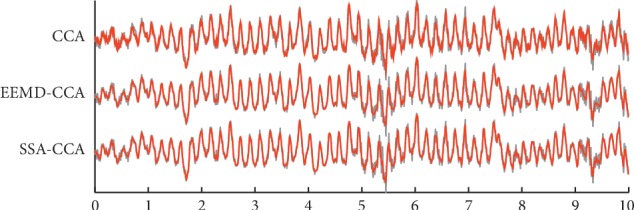
The denoised EEG (red) of channel T2 presented together with the raw EEG (grey) by applying (a) CCA, (b) EEMD-CCA, and (c) SSA-CCA. The horizontal axis is on behalf of time variation with the second as the unit.

**Figure 7:**
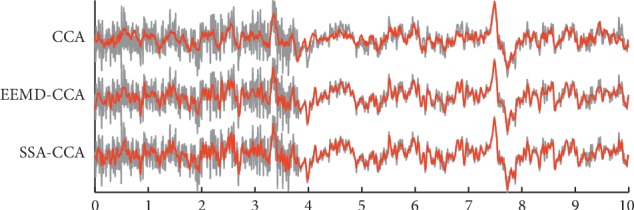
The denoised EEG (red) of channel T5 presented together with the raw EEG (grey) by applying (a) CCA, (b) EEMD-CCA, and (c) SSA-CCA. The horizontal axis is on behalf of time variation with the second as the unit.

**Figure 8:**
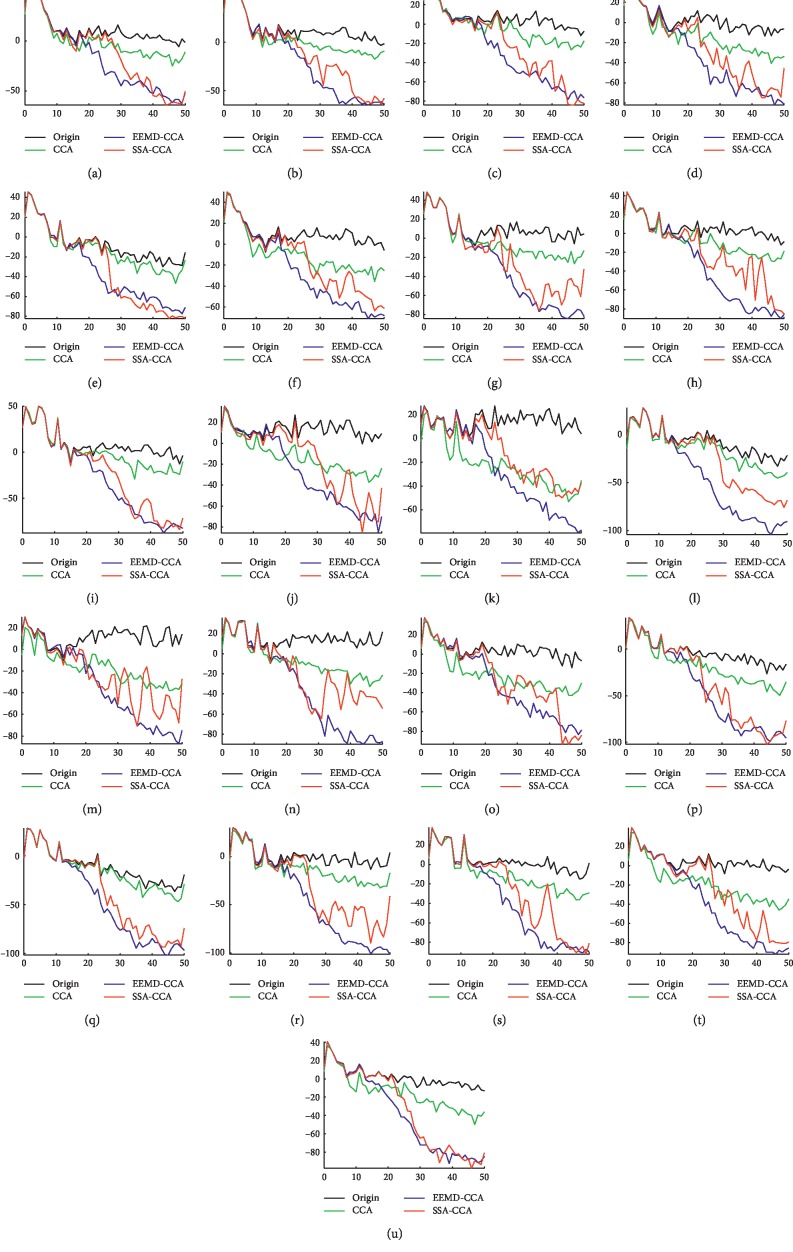
Power spectral density of the raw EEG and the denoised EEG processed by different methods in our study. The horizontal axis is frequency with unit Hz, and the vertical axis is PSD with unit dB: (a) Fp1; (b) Fp2; (c) F7; (d) F3; (e) Fz; (f) F4; (g) F8; (h) T1; (i) T2; (j) T3; (k) C3; (l) Cz; (m) C4; (n) T4; (o) T5; (p) P3; (q) Pz; (r) P4; (s) T6; (t) O1; (u) O2.

When it comes to the real data, without the ground truth, the two evaluation indexes of RRMSE and ACC cannot be employed to illustrate the denoising effects of these methods. Here, the comparative results on qualitative time domain waveforms and the PSD values before and after artifact removal are applied. In order to get a closer look, a channel lightly polluted and a channel heavily contaminated by muscle artifacts were picked out, they were T2 and T5, respectively. The temporal waveforms after applying three different methods to the real EEG are described in Figures 6 and 7. The grey color denotes the original real-life data, and the red color denotes the cleaned data. From Figure 6, EEMD-CCA and SSA-CCA could perfectly deal with lightly contaminated EEG since the artifacts appearing at around 5.3 s and 9.2 s were removed and the seizure activity was reserved very well. The resulting signal of CCA could also follow the original waveform, but the artifacts were not eliminated very cleanly. From Figure 7, when EEG was corrupted with heavy artifacts, it could be seen that there were still a lot of visible artifacts in the result of CCA. In addition, the cleaned signal failed to go after the raw signal at the segment free from artifacts between 4 s and 10 s. But EEMD-CCA and SSA-CCA can deal with this situation successfully. The denoised signals closely followed the raw signal segments where there were no artifacts and visible artifacts could not be found. By carefully examining the waveform details in Figures 6 and 7, we are able to conclude that SSA-CCA is more powerful than EEMD-CCA in preserving brain activity. There are more details in the waveform of SSA-CCA, meaning that more EEG information can be preserved. For further illustration of this point, we also computed the values of PSD for the raw EEG data and the artifact-attenuated EEG data processed by these three methods. The PSD values are plotted channel by channel in Figure 8.

As it can be seen in Figure 8, the EEG signals in channels Fz, Cz, and Pz are merely lightly polluted. Therefore, the PSD values (black) of these channels are relatively higher at low frequencies and lower at high frequencies. Noticing the characteristics of the spectrum distribution in Fz, Cz, and Pz, we can note that true EEG contents in the raw EEG are originally concentrated at low frequencies (e.g., 1–25 Hz). There are obvious EEG rhythms at about 10 Hz and between 1 and 5 Hz. For the channels polluted by heavy artifacts, such as T4, C4, T3, and C3, the PSD values at high frequencies are relatively higher. The goal of denoising is to maximally suppress the effects of muscle activity and meanwhile minimally cause a loss to brain activity.

From Figure 8, the performance of CCA is unsatisfactory. CCA does not have enough effect on removing muscle artifacts (e.g., F7 and P4), causing the insufficient energy decrease in the high-frequency band, or removes both brain and muscle contents, resulting in the energy decrease in almost all frequency bands (e.g., T3 and C4). This is because the sources decomposed by CCA are mainly the mixture of muscle artifacts and ongoing EEG signals. On the contrary, both EEMD-CCA and SSA-CCA can largely remove muscle artifacts, comparing the PSD values at high frequencies with those of the original EEG signal. And the PSD values between 1 and 5 HZ and around 10 Hz are nearly unchanged from the raw EEG signal in almost all channels, demonstrating the ability of retaining EEG content for the two methods. However, when observing the results between EEMD-CCA and SSA-CCA more closely, we are able to see that the PSD values in terms of EEMD-CCA decrease sharply from about 15 Hz while the PSD values in terms of SSA-CCA tend to keep relatively higher between 15 and 25 Hz in almost all channels. Thus, SSA-CCA can preserve EEG information not only in the low-frequency band but also in the relatively higher frequency band. To be more specific, in the frequency band of 15–25 Hz, the PSD values of SSA-CCA are very close to those of the original EEG signal with little contamination in channels Fz, Cz, Pz, T6, and O2, indicating that EEG information is well retained; meanwhile, taking the PSDs of the original EEG signal heavily contaminated in channels T3, C3, C4, and T4 as a reference, the PSD values of SSA-CCA are lower, indicating that muscle artifacts are removed. EEMD-CCA thoughtlessly ignores the brain activity in the frequency band of 15–25 Hz. Hence, SSA-CCA is more powerful than EEMD-CCA in extracting EEG information of higher frequency.

**Figure 9:**
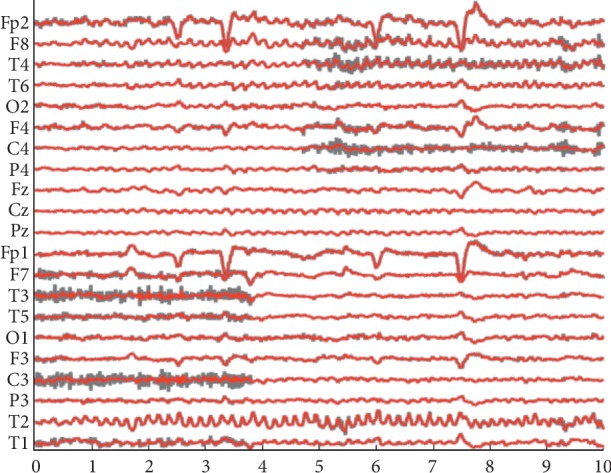
The reconstructed EEG (red) after eliminating EMG artifacts presented together with the raw EEG (grey).

In addition, by using the proposed SSA-CCA method, the final denoised EEG data are shown in Figure 9. As we can see, compared with the raw EEG data, muscle artifacts completely disappeared while the EEG content was well reserved.

## 5. Discussion and Conclusion

According to the relevant discussions in [26, 39], it is crucially important to guarantee the signal quality of EEG by means of muscle artifact removal. Different from ocular and cardiac artifacts, muscle artifacts with highly nonstereotyped scalp topographies are especially challenging to be eliminated. This may be the reason why ICA, a well-known and widely used tool, does not perform well for removing muscle artifacts. Although ICA has a good effect on ocular and cardiac artifact removal, based on previous studies [9, 13–17, 23, 26], there is no need to apply ICA for comparison in the multichannel EMG artifact removal task in this paper. Instead, CCA makes use of the unique characteristics of muscle activity such as low autocorrelation, resulting in an improved performance. However, the traditional multichannel BSS techniques, like CCA, are capable of extracting the underlying myogenic sources as many as the number of EEG channels at most. When the SNR is very low with complex and severe contamination, the potential sources might be more than utilizable channels in number. Under these circumstances, combining single-channel decomposition methods with BSS is recommended, for example, EEMD-CCA.

As we all know, the architecture of the human head is often regarded as a volume conductor, so that the interference of each muscle can readily appear anywhere on the scalp. Thus, the signal in each channel generates from the mixture of different underlying sources, bringing in crosschannel dependence. The main advantage of single-channel decomposition may be that this technique fully explores single-channel information to discover independent EEG sources. The subsequent division of sources in each channel reduces the complexity of the EEG signal, and the following BBS method is applied to conduct further noise reduction with the crosschannel information. The algorithm architectures of EEMD-CCA and SSA-CCA are different, but they both take advantage of combining single-channel decomposition with BSS. Since EEMD decomposes signals merely according to amplitude and frequency, the frequency spectrum of the artifact sources derived from EEMD often overlaps with that of EEG sources. The identified artifact-related IMFs absolutely contain EEG content, which is drowned in the artifacts. Even CCA cannot completely extract the EEG content from the artifact-related IMFs. The high-frequency EEG is mixed with the last sources abandoned by CCA. This explains why a lot of loss was caused by EEMD-CCA in the frequency band of 15–25 Hz processing the real data. However, taking advantage of the information of eigenvalues, SSA is able to distinguish diverse sources even mixed in the time-frequency domain. It can be seen that EEG-related RCs with low eigenvalues, accounting for a small portion of EEG contents, are nicely separated by SSA in Figure 4. The high-frequency EEG part is not dominant in the pure EEG; thus, it tends to be the EEG-related RCs with low eigenvalues and is well retained by SSA. As for muscle artifacts owning extensive frequency domain distributions, SSA can get better results than EEMD.

The window length *L* of SSA is selected according to the condition, i.e., *L* > *f*_s_/*f*_l_, where *f*_l_ is the lowest frequency of the desired component and *f*_s_ is the sampling frequency [40]. This choice can make sure that the size of *L* is large enough to include one period of the desired source at least. Therefore, we set the *L* to be 200 and 100 for semisimulated data and real data, respectively. In practical applications, the real data recordings are complicated and it is hard to select a proper window length. Fortunately, we tested different window length values (i.e., 200, 150, 100, and 50) with all other parameters unchanged. The results on semisimulated data and real data were very similar, all leading to advantages over EEMD-CCA. It demonstrates that in our proposed method, SSA is not sensitive to *L*, showing the generality of the method. We recommend choosing the window length *L* according to the condition *L* > *f*_s_/*f*_l_ if one wants to obtain a detailed decomposition by SSA. But in practice, *L* is proposed to be between 50 and 100, considering the good decomposition effect and low time cost. It must be mentioned that Maddirala et al. grouped the eigenvectors on the basis of the local mobility of the eigenvectors to eliminate muscle artifacts from EEG [41]. We also used the local mobility to distinguish RCs and found the results were totally consistent with the criterion of calculating the autocorrelation. In our proposed method, two thresholds of the autocorrelation coefficient should be determined, one for choosing EEG-related RCs and the other for CCA discarding artifact-related sources. In [26] for EEMD, to preserving EEG content as much as possible in the early processing step, the first threshold is relatively higher (0.95 used in our study) for selecting artifact-related IMFs. While in our proposed method, the first threshold is set to be lower, in order to pick out EEG-related RCs as possible. Through experiments, the thresholds around 0.8 are recommended (0.82 used in our study). In the semisimulation study, with ground truth, the optimally selected number of abandoned components can be determined for all the methods, thus the second threshold is not needed. In the real-life data study, the second threshold values ranged from 0.80 to 0.99 were examined through direct visual inspection of both temporal and spectral contents before and after muscle artifact elimination. All the methods adopt 0.9 as the second threshold for a fair comparison.

For practical applications, we tested the time cost of conducting CCA, EEMD-CCA, and SSA-CCA. In SSA, calculating the eigenvalues of the trajectory matrix is time-consuming work. On the premise of not affecting the effect of denoising, *L* was set to be 50 for comparison. The realization was completed in MATLAB (MathWorks Inc., Novi, MI, USA) and operated under Microsoft Windows 10 × 64 on a computer with Intel(R) and Core(TM) i5-8400 2.80 GHz CPU and 16.0 GB RAM. At each SNR from 0.5 to 4.5 changing with a 0.5 step, there were 200 independent implementations for semisimulated data mentioned above. The average time costs for CCA, EEMD-CCA, and SSA-CCA over the EEG data with 19 channels and the length of 10 seconds were 0.0118 s, 4.4469 s, and 3.6665 s with standard deviations 0.0007, 0.1529, and 0.0218. As you can see, the computational cost of SSA-CCA can be completely accepted.

We have proved that SSA-CCA is a satisfactory tool for muscle artifact elimination when processing the multichannel EEG signals. Nowadays, with portable or wearable EEG devices for long-term mobile monitoring becoming increasingly prevalent, the present EEG devices have a tendency to own only a small number of channels [18]. Unfortunately, our method might not be the optimal choice due to the channel limitation in the few-channel situation [39]. Thus, we might improve the current processing architecture of SSA-CCA to satisfy the needs of removing muscle artifacts from few-channel EEG signals in the near future.

## Data Availability

There are two data sets used, semisimulated data and real-life data. The semisimulated data used to support the findings of this study are available from the authors Qingze Liu and Aiping Liu upon request, who can be contacted via qingze@mail.ustc.edu.cn and aipingl@ece.ubc.ca, respectively. The real-life data are publicly available from the BioSource database, and one can access this data set at “http://www.esat.kuleuven.be/sista/members/biomedng/biosource.html.”
